# Retraction of “Photoelectrochemical Synthesis of Benzo[*b*]phosphole Oxides via Sequential P–H/C–H Bond Functionalizations”

**DOI:** 10.1021/acscatal.5c03083

**Published:** 2025-05-19

**Authors:** Nayan Saha, Burkhard König

The authors retract this article (DOI: 10.1021/acscatal.4c06292) due to the fact that the Nuclear Magnetic Resonance (NMR) spectra reported in the Supporting Information and raw data FID files were edited to obscure impurities in the analyzed samples. On April 7, 2025, a Correction (DOI: 10.1021/acscatal.5c01983) was published to correct the appearance of NMR spectra in the Supporting Information and add a hyperlink to the raw NMR FID files. However, following this Correction, additional concerns were raised with regard to postcollection editing of the spectra to obscure impurities. The compounds whose spectral data were altered are central to the conclusions of the work; as such, this article is being retracted.

*The original article was published on November, 20, 2024, and was retracted on May 19, 2025.*

